# Effect of predrying treatment and drying temperature on proximate composition, mineral contents, and thermophysical properties of anchote (Coccinia abyssinica (Lam.) Cogn.) flour

**DOI:** 10.1002/fsn3.1860

**Published:** 2020-08-31

**Authors:** Adugna Mosissa Bikila, Yetenayet Tola, Tarekegn Berhanu Esho, Sirawdink Fikreyesus Forsido

**Affiliations:** ^1^ Jimma University College of Agriculture and Veterinary Medicine Jimma Ethiopia; ^2^ Addis Ababa Science and Technology University Addis Ababa Ethiopia

**Keywords:** anchote, drying, nutrient content, pre‐drying treatment, thermophysical property

## Abstract

Anchote (Coccinia abyssinica (Lam.) Cogn.) is an indigenous underutilized but valuable root crop solely consumed in Ethiopia. It is commonly used as occasional food during holidays and as therapeutic food to treat fractured bone or develop strong bone. So far, different studies conducted related to its variety development, nutrition, and anti‐nutrition contents. In this study, attempts were made to investigate the effect of predrying treatments and drying temperature on nutritional composition and thermophysical properties of its flour. A general full factorial experiment with two factors each at three levels used to run the experiment. The proximate compositions, thermophysical properties, and mineral contents were determined using AOAC standard methods; well‐developed model equations; and atomic absorption spectrophotometry, respectively. The ranges of nutrients in mg/100 g DB were as follows: moisture content (MC) 7.37–10.10; protein 3.26–4.80; total fiber (TF) 7.24– 10.76; ether extract (EE) 0.49–0.70, ash 2.96–4.74, total carbohydrate (TC) 80.01–83.66; calcium (Ca) 357.07–482.89; magnesium (Mg) 138.87–222.38; iron (Fe) 10.87–16.00; zinc(Zn) 2.07–2.80; and copper(Cu) 1.90–2.25. The bulk density, specific heat capacity, thermal conductivity, and thermal diffusivity of the flours ranged in 0.72–0.82 (g/cm^3^), 1.710–1.780 KJ/kg°C, 0.263–0.273 W/m^2^°C, and 1.88 x 10–4– 2.12 × 10–4 m^2^/s, respectively. The predrying treatment and drying temperature significantly affected (*p* < .05) MC, protein, TF, EE, ash, TC, Ca, Fe, and Zn contents. The thermophysical properties were also affected by the same factors. The flour made from raw anchote dried at 100°C was better in preserving the nutrients and improving its cooking property.

## Graphical abstract

Anchote tuber flour obtained from raw, blanched, and boiled samples after drying at temperatures of 60, 80, and 100°C were evaluated for their nutritional values and thermophysical properties. The result showed that the predrying treatment and drying temperature significantly (*p* < .05) affected MC, protein, TF, EE, ash, TC, Ca, Fe, and Zn contents as well as thermophysical properties of the samples. The flour obtained from the raw tuber dried at relatively higher temperature (up to 100°C) could be recommended for better nutritional value and thermal property.

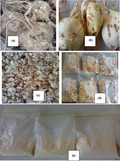

## 1 INTRODUCTION

**FIGURE 1:**
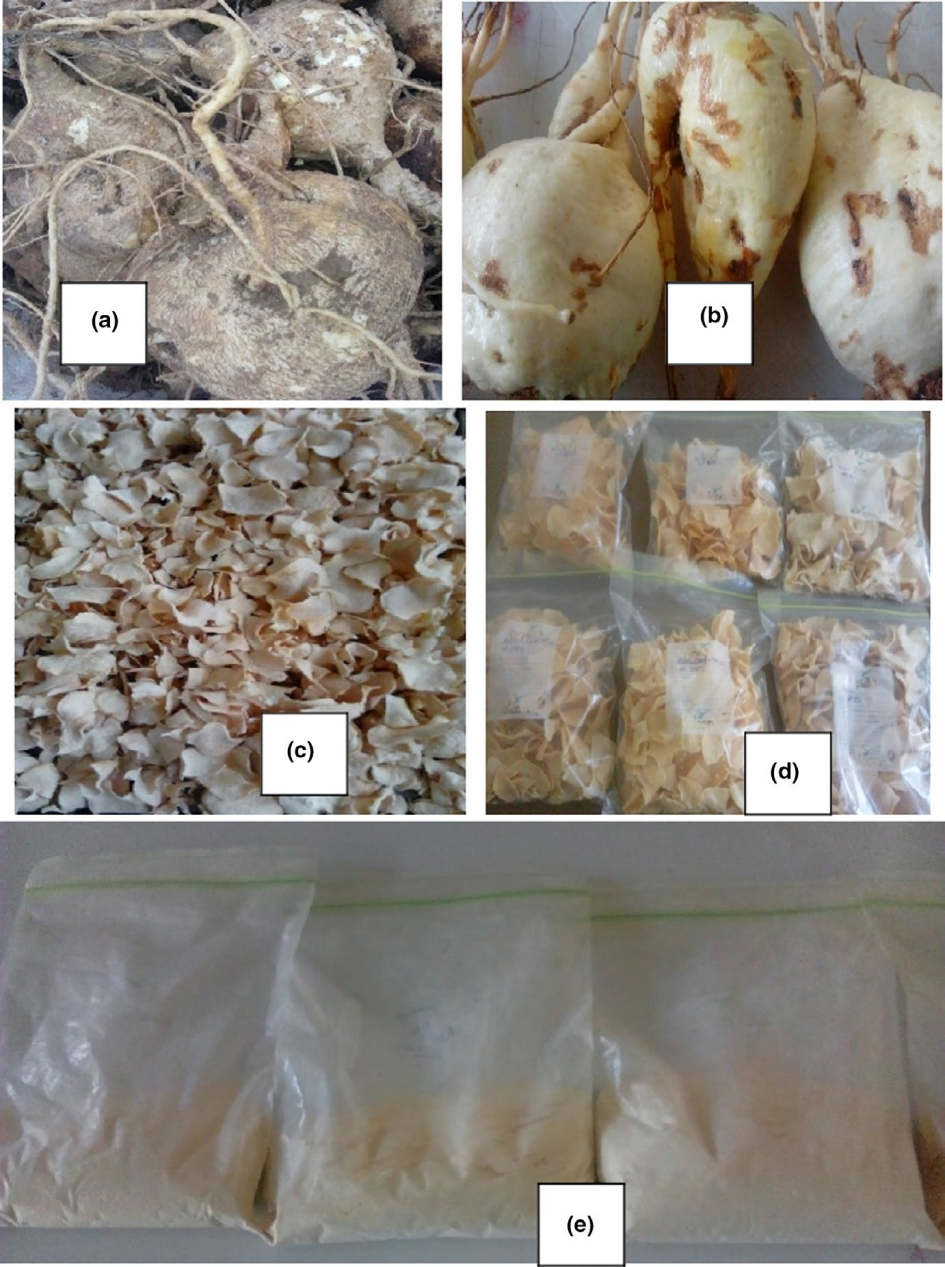
Anchote tuber and its processed products (a)—fresh anchote tuber, (b)—washed anchote tuber, (c)—dried tuber flake, (d)—packed flake, (e)—packed anchote flour)

Anchote (*Coccinia abyssinica (Lam.) Cogn*.) is a potentially productive and nutritious tuberous crop endemic to Ethiopia. The term “Anchote” is an Afan Oromo common name spelled as “Ancootee.” It was originating particularly in East Wollega Zone of Oromia Region in South West of the country (Fekadu, 2014). The crop is relatively new to the rest of the country and to the world with limited scientific information (Tolera, 2017). It is the first time the first improved Anchote variety *code 25 (Desta1)* released by Debrezeit Agricultural Research Center of the Ethiopia Institute of Agriculture Research (*DZARC/EIAR*) in 2018. The freshly harvested and washed anchote (*Coccinia abyssinica (Lam.) Cogn*.) tuber is shown in Figure 1 A,B. When compared to other common root and tuber crops, anchote is highly nutritious and also has therapeutic advantages and social values (Abera & Haile, 2015; Parmar et al., 2017). Literature data indicated that its tuber contains valuable nutrients such as carbohydrate, protein, fiber, fat, and different minerals (Ayalew, 2016; Fekadu, 2014; Parmar et al., 2017). Notably, the tuber is unique in its high protein and calcium content, which are usually low in other tuberous crops.

Processing and value addition of raw food materials of plant origin are an essential step to bring stability and reduce postharvest losses of the tuber. In Wollega zones, anchote stew (*locally called “ittoo ancootee”*) is a popular processed product (boiled tuber) available with injera in some restaurants (Fufa & Urga, 1997; Parmar et al., 2017). However, under conventional cooking practice, the tuber is hard to cook and takes a long time (up to 3 hr) when compared to other starchy tubers such as cassava and sweet potato (Parmar et al., 2017), which results in need of more fuel wood as energy source to cook. This might be one of the limitations for the extensive utilization of the tuber in different forms and different parts of the country and elsewhere. Apart from high energy demand, boiling for an extended time may affect the nutrient composition of the tuber due to heat degradation and leaching out in boiling water. Furthermore, like other tuber crops, the tuber of anchote cannot be stored for an extended period due to its perishable nature and loss in quality. Commonly underground storage practiced to avoid after harvest losses, but this inhibits producers not to use the same field for other crops.

In addition to the traditionally cooked tuber by boiling, certain trials were made to develop value‐added food products, such as biscuit and bread, using anchote tuber as an ingredient (Haile, Bekele, & Desalegn, 2018; Hailu, 2016; Shebabaw, 2013). However, the combined effect of predrying treatment of the tuber (blanching and boiling) and drying temperature on nutritional composition and thermophysical property of anchote flour not yet studied. We presumed that both predrying treatment and drying temperature could affect nutritional composition and thermal and physical properties of the tuber flour as compared with the raw. Consequently, it is necessary to explore available preservation and value‐added option to overcome the above limitations and further commercialization of the tuber products to other parts of the country. For instance, the flour made from the tuber not only helps to overcome the limitations but also can be used as an ingredient to formulate a variety of products for different social groups. Therefore, this study aimed at investigating the effect of predrying treatments and drying temperatures on proximate compositions, mineral contents, and thermophysical properties of anchote flour as a value‐added product.

## 2 MATERIALS AND METHODS

### 2.1 Materials

The plant material used for this study was anchote variety of code 25 (*Desta1*). It is the first improved variety released by Debrezeit Agricultural Research Center of the Ethiopia Institute of Agriculture Research (*DZARC/EIAR*) in 2018.

### 2.2 Research design

A general factorial experiment in a completely randomized design employed to run the experiment. Predrying treatment having three levels (raw untreated (control), blanching, and boiling) included in the first factor, followed by drying temperature of the slice in three levels (60, 80, and 100°C) by replicating each treatment combination three times.

### 2.3 Sample preparation

Anchote tuber washed in running tap water to remove the adhering substances, peeled off using stainless steel knives, washed farther with tap water, and sliced into ~2 mm thickness. The homogenized sample then grouped into three: raw tuber, tuber subjected to blanching in hot water (95–100°C) for 5 min, and tuber boiled in boiling water for 30 min. The samples then dried in an oven (*LABQUIP, LEICESTER LE67 5FT, England*) at temperatures of 60, 80, and 100°C till to no difference in measurement between two consecutive weight measurements. Dried tuber flake (Figure 1c,d) then ground into flour (Figure 1e), sieved through a 500‐μm mesh screen, packed in moisture‐proof materials, and stored at −4°C until analysis.

### 2.4 Data collected

#### 2.4.1 Proximate composition

##### Moisture content

The moisture content of the flour determined according to the AOAC (2010) official method number 925.10. Briefly, 2 g sample was weighed in previously heated at 130 ± 3°C, cooled, and weighed dish. Then, the dish and its content were heated in the oven (*LABQUIP, LEICESTER LE67 5FT, England*) at 130 ± 3°C for 1 hr. Finally, cooled in a desiccator to room temperature and weighed; the moisture content then obtained from the weight loss by difference.

##### Crude protein content

Protein content was determined using the automatic *Velp Scientifica Kjeldahl* analyzer instrument (*UDK 159*), according to AOAC Official Method 920.87 (2010). Briefly, anchote flour sample (1.0 g) mixed with a catalyst (*K_2_SO_4_ and CuSO_4_.5H_2_O*) and digested in 12 ml concentrated sulfuric acid at 420°C for 60 min to liberate the organically bound nitrogen in the form of ammonium sulfate. The ammonia in the digest ammonium sulfate then distilled off with reagents (*50 ml distillation water, 50 ml NaOH, and 30 ml H_3_BO_3_*) added and then automatically titrated with standard hydrochloric acid (0.2 N). Finally, a conversion factor of 6.25 used to convert from total nitrogen content to determine crude protein of the samples.

##### Fiber content

Crude fiber content was determined by the Weende scheme as adopted by Bekele and Bekele (2018). About one gram of the dried sample was boiled for 40 min in 50 ml dilute sulfuric acid (2.5%) and filtered. The residue then washed with distilled water and again boiled in 50 ml of 2.5% sodium hydroxide for 40 min. The residue consecutively washed with 20 ml ethanol (99.8%) and 20 ml diethyl ether two times, followed by washing with 20 ml acetone three times. The insoluble residue consisted of crude fiber, and ash was dried in oven (Blast Air Oven, DHG‐9240A, China) and weighed (w_1_). This residue was burned in a furnace (Nabertherm, D‐6072 Dreieich, Germany) at 550°C for 3 hr (w_2_) and the weight difference (w_1_‐w_2_) taken as crude fiber.

##### Ether extract

The ether extract (taken as crude fat content) in the sample was determined by the soxhlet (Diethyl ether) method, as described by Bekele and Bekele (2018). The dried anchote flour sample extracted with diethyl ether for 1 hr. Then, the extract was dried in an oven (Blast Air Oven, DHG‐9240A, China) at 105°C for 3 hr. This ether extract was weighed and taken as crude fat content.

##### Ash content

The ash content of the flours was determined using AOAC (2010) method number 923.03. A 2 g sample was weighed in ignited, cooled, and previously weighed ashing dish and ignited in a furnace (CARBOLITE, S33 6RB, England) at 550°C until ash results, and the sample then placed in desiccators to room temperature and weighed. The remaining weight in the dish was determined by difference and taken as ash content.

##### Total utilizable carbohydrate

The total utilizable carbohydrate content of the samples was determined by difference (Nascimento & Canteri, 2018).

(1) $$\%\text{Carbohydrate}\;\;\text{content}=100\text{\textbackslash{}\%}\;‐\left(\text{\textbackslash{}\%}\;\;\text{moisture}+\text{\textbackslash{}\%}\;\;\text{protein}+\text{\textbackslash{}\%}\;\;\text{Fat}+\text{\textbackslash{}\%}\;\;\text{Ash}\right)$$

##### Energy value

The energy value (Kcal) of each sample was determined according to the already established Atwater factor method, as described by Lewu, Adebola, and Afolayan (2009).

(2) $$E.V=\left(9\;x\;\text{Crude}\;\text{Fat}\;\text{\textbackslash{}\%}\;\right)+\left(4\;x\;\text{Crude}\;\text{Protein}\;\text{\textbackslash{}\%}\;\right)+\left(4\;x\;\text{Carbohydrate}\;\text{\textbackslash{}\%}\;\right)$$

#### 2.4.2 Mineral analysis

For the determination of some minerals (Ca, Mg, Fe, Zn, and Cu), a 1.0 g anchote tuber flour was calcinated in a muffle furnace (Nabertherm, D‐6072 Dreieich, Germany) at 450°C for 4 hr. The ash was dissolved in 20% nitric acid and filtered using the Whatman filter paper (42 mm) before analysis. Then, the minerals Ca, Mg, Fe, Zn, and Cu were determined using atomic absorption spectrophotometer (SHIMADZU, AA‐6880F, Japan) using air/acetylene flame at wavelengths of 422.7, 285.2, 248.3, 213.9, and 324.7 nm respectively. Finally, the concentrations of the elements in the samples were calculated using the following equation.

(3) $$\text{Concentration}\;\left(\text{mg}/100g\right)=\frac{\left(a‐b\right)xV}{10xWs}$$

where a = concentration in ppm (mg/L) of sample; b = concentration in ppm (mg/L) of blank solution, V = Volume in ml of extract, Ws = Weight of sample in g, 10 = conversion factor.

#### 2.4.3 Determination of thermal and physical properties

##### Bulk density

Bulk densities of the flour samples were determined by a method used by Oluwole, Abosede, and Olushola (2016). Samples of 10 g (triplicates) measured into a 50 ml graduated measuring cylinder and gently tapped on the bench ten times to attain a constant height. The volume of the sample was recorded and expressed as grams per cubic centimeter (g/cm^3^).

##### Specific heat capacity (Cp)

The specific heat capacity was derived from the proximate composition of the samples as per the method described by Barine and Victor (2016) (Equation 4).

(4) Cp=4.180Xw+1.711Xp+1.929Xf+1.547Xc+0.908Xa

where Cp is specific heat capacity in *KJ/Kg K*, and *Xs* is the respective mass fractions of water (*Xw*), protein (*Xp*), fat (*Xf*), carbohydrate (*Xc*), and ash (*Xa*) present in each sample and obtained from proximate compositions.

##### Thermal conductivity (K)

The thermal conductivities of the samples were determined using the expression reported by Barine and Victor (2016) from the determined proximate composition (Equation 5).

(5) K=0.25Xc+0.155Xp+0.16Xf+0.135Xa+0.58Xw

where *K* is thermal conductivity of the sample in W/m^o^C, and *Xs* is the respective mass fractions of carbohydrate (Xc), protein (Xp), fat (Xf), ash (Xa), and water (Xw) present in each sample.

##### Thermal diffusivity (α)

The method described by Nwanekezi and Ukagu (1999) was used to determine the thermal diffusivity of the flour samples, as indicated in Equation 6.

(6) $$=\frac{k}{\text{PCp}}\left(m^{2}/s\right)$$

where K = thermal conductivity (Wm^‐1o^C^‐1^), *ρ* = bulk density (kgm^‐3^), and Cp = specific heat capacity (kj/kg ^o^C).

### 2.5 Statistical analysis

The data then subjected to analysis of variance (*ANOVA*), and the significance of the difference between means determined at a 95% confidence level (*p *≤ .05) using Minitab (Version 16, USA) with Tukey's method. The result values were expressed as means of triplicates ± standard deviations.

## 3 RESULTS AND DISCUSSION

### 3.1 Proximate composition

#### 3.1.1 Moisture Content (MC)

The effect of predrying treatments (blanching and boiling) and drying temperature on the MC of anchote flour is presented in Table 1. The MC ranges between 7.1 and 10.40 g/100 g for all the test samples. As expected, an increase in drying temperature significantly (*p* < .05) decreased MC of the samples except flours made from boiled anchote tuber. When MC values at the same temperature compared, at 60°C no significant (*p* < .05) difference observed among the three pretreatment methods. The same was observed at 80°C for raw and blanched samples; however, a relatively higher value of MC obtained for flour obtained from boiled tuber. At 100°C, significant effects of the pretreatments were observed with the highest value for the flour of boiled tuber, followed by blanched and raw tubers. This implies that thermal treatment might modify the physicochemical properties of the tuber starch and might alter its water holding capacity as compared to flour of the raw sample. Our result in this study was relatively higher than the MC of raw (3.25%) and blanched (4.48%) potato flours (Nascimento & Canteri, 2018). An increase in MC after boiling was in agreement with that observed in *Dioscorea dumetorum* flour as indicated in Ezeocha, Ojimelukwe, and Onwuka (2012).

**TABLE 1:** Proximate composition of raw, blanched, and boiled Anchote flour dried at different temperatures

PT	DT (^o^C)	Proximate composition (g/100g DW)						EV (Kcal)
		MC	CP	TF	EE	Ash	CH	
Raw	60	10.10 ± 0.20^a^	4.54 ± 0.18^ab^	7.54 ± 0.78^bc^	0.70 ± 0.01^a^	4.64 ± 0.06^b^	80.01 ± 0.13^e^	344.54 ± 0.59^e^
	80	9.10 ± 0.17^b^	4.29 ± 0.15^bc^	8.22 ± 1.40^b^	0.60 ± 0.01^d^	4.74 ± 0.06^a^	81.26 ± 0.11^d^	347.62 ± 0.91^de^
	100	7.37 ± 0.24^d^	3.76 ± 0.35^d^	7.57 ± 0.51^bc^	0.53 ± 0.02^e^	4.69 ± 0.07^ab^	83.66 ± 0.38^a^	354.39 ± 0.91^ab^
Blanched	60	9.75 ± 0.20^a^	4.49 ± 0.10^b^	7.63 ± 1.27^bc^	0.53 ± 0.03^e^	3.68 ± 0.08^c^	81.57 ± 0.22^d^	348.93 ± 0.74^d^
	80	8.82 ± 0.34^bc^	4.80 ± 0.23^a^	10.76 ± 0.15^a^	0.52 ± 0.02^e^	3.61 ± 0.01^cd^	82.26 ± 0.22^c^	352.89 ± 1.35^b^
	100	8.12 ± 0.12^c^	4.13 ± 0.65^c^	10.58 ± 0.26^a^	0.54 ± 0.04^e^	3.64 ± 0.04^c^	83.57 ± 0.68^ab^	355.67 ± 0.40^a^
Boiled	60	9.80 ± 0.88^a^	3.25 ± 0.24^e^	7.24 ± 0.33^c^	0.67 ± 0.02^b^	3.12 ± 0.08^cd^	83.16 ± 0.57^b^	351.63 ± 3.30^bc^
	80	10.10 ± 0.25^a^	3.42 ± 0.22^e^	10.16 ± 0.41^a^	0.49 ± 0.01^e^	2.96 ± 0.04^e^	83.06 ± 0.34^b^	350.04 ± 1.08^d^
	100	10.10 ± 0.25^a^	3.26 ± 0.33^e^	10.05 ± 0.28^a^	0.63 ± 0.04^c^	2.96 ± 0.02^e^	83.06 ± 0.24^b^	350.89 ± 1.02^cd^
LSD (0.05)		0.362	0.309	0.730	0.024	0.055	0.367	1.386
CV (%)		3.9	7.8	8.3	4.2	1.5	0.5	0.4

Note

Data are mean ± standard deviation of triplicate determinations. Means with same letters in a column are not significantly different (*p* < .05)

Abbreviations: CH, carbohydrate; CP, crude protein; CV, coefficient of variation; DT, drying temperature; EE, ether extract; EV, energy value;LSD, least significant difference; MC, moisture content; PT, predrying treatment; TF, total fiber.

When compared to other root and tuber crops, the average MC of raw anchote (8.86 g/100 g) is similar to taro flour (8.19 g/100 g) reported by Aprianita, Purwandari, Watson, and Vasiljevic (2009). Nevertheless, it is higher than the MC of sweet potato and potato tubers flours that were 7.07 and 3.25 g/100 g, respectively (Aprianita et al., 2009; Nascimento & Canteri, 2018); and it is in close agreement to yam flour (10.51%) as reported in Aprianita et al. (2009). The MC of blanched anchote flour (8.89 g/100 g) was comparable with that of blanched yellow sweet potato flour (8.72 g/100 g) (Nogueira, Sehn, Rebellato, & Coutinho, 2018), but higher than blanched potato flour (4.48 g/100 g) (Nascimento & Canteri, 2018). Results in this work indicate that the MC content of the flours from all the test combinations is lower than the recommended moisture content (12%–13%) for excellent storage stability of flours (Chukwu & Abdullahi, 2015). Therefore, anchote flour can be safely stored for extended time utilization.

#### 3.1.2 Crude protein (CP)

The average protein contents of raw, blanched, and boiled anchote flours dried at different temperatures are presented in Table 1. The results showed that CP of the raw and blanched anchote flours was not significantly different (*p* > .05). However, it was reduced significantly in the boiled sample. The decrease in CP after boiling was about 21.2%, which is in agreement to the report of Fekadu (2014). The decreasing trends of CP content after blanching and boiling of different root and tuber crops indicated in different works (Aathira & Siddhuraju, 2017; Adane, Shimelis, Negussie, Tilahun, & Haki, 2013; Ezeocha & Ojimelukwe, 2012). The reduction could be associated with the loss due to water solubility of proteins during blanching and boiling (Ukom, Richard, & Abasiekong, 2018). The effect of drying temperature on the protein content of the flour samples was also significant (*p* < .05). The raw and blanched anchote dried at 60 and 80°C preserved more protein than other treatments. However, the protein content decreased with an increase in drying temperature except for the boiled tuber. A similar result also observed in orange‐fleshed sweet potato tuber (Haruna, Adejumo, Chinma, Akanya, & Okolo, 2018). Thermal denaturation of proteins might be the possible reason for the reduction of CP content with an increase in drying temperature (Qixing et al., 2014). Even the predrying treated and dried anchote flour contain relatively higher protein than widely consumed root and tuber crops such as sweet potato (3.40%), cassava (1.5%), and potato (1.70%) (Moongngarm, 2013; Nascimento & Canteri, 2018). However, it was lower compared to water yams (purple 6.84 and yellow 5.62%) and taro (6.43%) (Adane et al., 2013; Harijono, Saputri, & Kusnadi, 2013). From the result, we can conclude anchote flour from raw or blanched tuber dried at 60°C can be used as a protein source to replace other root and tuber crops, which are commonly deficient in protein content.

#### 3.1.3 Total fiber (TF)

High fiber content of foods is beneficial in promoting various physiological and metabolic effects by controlling the absorption of unhealthy food components and reducing oxidative processes (Aathira & Siddhuraju, 2017; Baah, Maziya‐Dixon, Asiedu, Oduro, & Ellis, 2009). It reduces blood cholesterol and glucose (Aathira & Siddhuraju, 2017), aids digestion, and protects the body against colon cancer, diabetes, and cardiovascular illnesses (Egbuonu & Nzewi, 2016). As indicated in Table 1, the TF content of anchote flour increased by predrying treatments (blanching or boiling) at relatively higher drying temperatures. The TF content was higher in the blanched and boiled samples dried at 80 and 100°C. The effects of both drying temperature and predrying treatments on the fiber content were statistically significant (*p* < .05). The result showed that pretreatment and higher temperature drying enhanced the TF content, which is in agreement with the report of Lewu et al. (2009) for potato and cocoyam flours, whose fiber content, respectively, increased from 1.64% to 1.66% and 1.83 to 2.49% by boiling. The increase might be due to gelatinization and retrodegradation of the starch as a result of heat treatment. Furthermore, the temperature could modify the native starch into nondegradable polysaccharides (resistant starch) that adds weight on fiber (Lewu et al., 2009). Both blanching and boiling might enhance the loss of water‐soluble components and increase the percentage of macromolecules rich in dietary fiber. The TF of the raw and pretreated anchote flours (6.34 – 10.92 g/100 g) was higher than values reported for other tuberous crops (potato, sweet potato, cassava, cocoyam, and yam) by several previous researchers (Aathira & Siddhuraju, 2017; Baah et al., 2009; Bekele & Bekele, 2018; Egbuonu & Nzewi, 2016; Harijono et al., 2013; Lewu et al., 2009; Nascimento & Canteri, 2018; Onodu, Culas, & Nwose, 2018; Ukom et al., 2018). Therefore, anchote flour can be used to enhance dietary fiber by incorporating into other foods or by aiding in the formulation of diets for people with diabetes and other health‐conscious individuals.

#### 3.1.4 Ether extract (EE)

Root and tuber crops generally known for their low fat content, and the same also observed in anchote with EE content of less than 1%. The average EE (taken as crude fat) of the raw and pretreated anchote tuber flours dried at different temperatures ranged in 0.49–0.70 g/100 g as presented in Table 1. The lower EE considered as a real opportunity for more extended storage stability of the flour due to less or no lipid oxidation problems to affect the flour flavor. Even though the total EE content is low, a significant (*p* < .05) difference in the EE contents observed among treatment combinations. The reduction in EE by blanching was in agreement with the report of Nascimento and Canteri (2018) in potato flour (raw 0.69% and blanched 0.62%). Ukom et al. (2018) also reported similar observations in cocoyam flour (unprocessed 1.20%, blanched 1.09%, and boiled 0.45%). The EE of raw anchote flour has shown a decreasing trend with increasing drying temperature. However, for the blanched flour, the change as a function of drying temperatures was not significant (*p* > .05). On the other hand, the EE of boiled anchote flour significantly increased with drying temperature (*p *< .05). This variation could be due to increased extractability of the more polar lipids that are bound to other macroconstituents of the tissue in the cooked samples (Lewu et al., 2009). The finding in this study indicated that the flour obtained from the raw anchote tuber dried at lower temperatures has preserved more fat.

#### 3.1.5 Ash

Ash is an indication of the total mineral content of a food material. The mutual effect of predrying treatment and drying temperature on the ash content of anchote flour is presented in Table 1. The result has shown that the effect of predrying treatment on ash content of the samples was significant (*p* < .05), unlike to drying temperature. The ash composition has shown decreasing trends by the predrying treatments when we move from raw to boiling pretreatment. Blanching reduced the ash content as compared to the raw (4.69 to 3.64 g/100 g) by 22.4%. A reduction in the ash content of different tubers after the blanching process is reported by different authors (Aathira & Siddhuraju, 2017; Fekadu, 2014; Harijono et al., 2013). Boiling also further reduced the average ash content of anchote flour (4.69 to 3.01 g/100 g) by 35.8% as compared to the raw. The same trends were reported for boiled *Dioscorea bulbifera*, *Dioscorea dumetorum*, and cocoyam tubers flours (Aathira & Siddhuraju, 2017; Ezeocha et al., 2012; Ukom et al., 2018). The reduction could be associated with leaching out of the minerals into the water (Ezeocha et al., 2012; Ukom et al., 2018) with the presence of heat, which harms mineral contents of the flour. The anchote flour obtained from the raw sample resulted in higher ash content, and this is an indication that more minerals preserved.

#### 3.1.6 Carbohydrate

Carbohydrate is among the major nutrients in root and tuber crops (Obadina, Ashimolowo, & Olotu, 2016 *)*. The level of total carbohydrate (TC) in anchote flours as affected by predrying treatment (blanching and boiling) and drying temperature is presented in Table 1. Both predrying treatment and drying temperature (*p* < .05) significantly affected the TC content of anchote flour. The TC has increased with increasing drying temperature, similar to that reported in orange flesh sweet potato dried at five temperature levels (Haruna et al., 2018 **).** Blanching and boiling also enhanced TC level of the flour, which is in agreement with the findings of Shebabaw (2013) in which carbohydrate content of two anchote accessions increased by boiling (Nekemte 77.31 to 78.72%, Dembidolo 77.95 to 79.35%). However, our result disagrees with the finding of Fekadu (2014) where a decrease of carbohydrate reported by boiling anchote. Increased carbohydrate content by blanching and boiling also recorded in other root and tuber crops such as *Dioscorea bulbifera, Dioscorea dumetorum,* Cocoyam, and bitter yam flours (Aathira & Siddhuraju, 2017; Egbuonu & Nzewi, 2016; Ezeocha & Ojimelukwe, 2012; Ukom et al., 2018). Generally, anchote flour obtained from the raw tuber dried at high temperature (100^o^C) provided higher TC content. Because the flour contained a high amount of carbohydrate, it can be used as an alternative source of energy.

#### 3.1.7 Energy value (EV)

The average EV of anchote flours ranged in 344.54–355.67 kcal as presented in Table 1. The EV per gram of the flour was significantly (*p *< .05) increased due to blanching and boiling. From the observed results, the variation is inversely related to the moisture content of the flours. This is in agreement with observations reported in tuber flours of cocoyam, potato, *Dioscorea bulbifera,* and *Dioscorea dumetorum* (Aathira & Siddhuraju, 2017; Ezeocha et al., 2012; Lewu et al., 2009). The effect of drying temperature also showed statistical significance (*p *< .05). The predrying treatment and increased drying temperature enhanced the EV. Of course, the effect is the reflection of carbohydrate, protein, and fat contents (Table 1). The average EV for raw anchote flour was similar to the report of Berhanu, Fikre, and Haile (2015), whereas lower compared to the report of Hailu (2016) (406.19 kcal). When compared with other tuber crops, the result was comparable with the EV of potato as reported by Nascimento and Canteri (2018). Though the comparison of EV for blanched and boiled samples not reported in anchote, similar data were reported for other roots and tuber crops. Nascimento and Canteri (2018) reported 361.90 kcal for raw and 367.21 kcal for blanched potato flour. Lewu et al. (2009) also recorded 376.30 and 384.66 kcal for raw and boiled potato, respectively. According to this finding, higher EV could be obtained from anchote flour by blanching/boiling followed by drying at higher temperatures (350.89–355.67 Kcal). This is in proportion with the carbohydrate composition because it contributes the highest share to calorific value of the flour.

### 3.2 Minerals content

#### 3.2.1 Calcium (Ca)

Calcium is very important in human nutrition for its function in blood clotting, muscle contraction, neurological functioning, bone and teeth maintenance, and enzyme metabolic processes (Sanoussi et al., 2016). The Ca content of raw and predrying treated anchote tuber flour dried at various temperatures presented in Table 2. Its average values were found in the range of 357.07–482.89 mg/100 g for all the treatments. The results showed that both the pretreatments (blanching and boiling) have significantly affected (*p *< .05) the Ca content of the flour by reducing the Ca content. A similar finding was reported by Shebabaw (2013) for anchote tuber taken from Nekemte accession (raw 470.13 and boiled 464.12 mg/100 g). This reduction could be due to the leaching of the mineral into the cooking water. On the other hand, increasing drying temperature has little effect on the Ca content of the flours. The level of Ca found in the anchote variety was greater than the report of Fekadu (2014) in which mean calcium content in raw and boiled anchote tuber was 119.5 and 115.70 mg/100 g, respectively. On the other hand, a very high Ca was recorded in Dembidollo anchote accession (raw 549.00 and boiled 544.30 mg/100 g) by Shebabaw (2013). The variations may be due to variety, growing ecology, tuber maturity, and treatment methods used (Abera & Haile, 2015; Ayalew, 2016; Shebabaw, 2013). It was observed that the anchote flour contains reasonably higher Ca compared to other common tuberous flours such as sweet potato (50.28–110.53 mg/100 g), potato (9 mg/100 g), and yam (31.02–118.8 mg/100 g) (Bekele & Bekele, 2018; Koua, Zoué^1^, T. L., Mégnanou, R. M., & Niamké, S. L., 2018; Rahman, Ali, Hasan, & Sarker, 2015). Generally, the raw anchote flour retained more Ca compared to the flour obtained from blanched and boiled tubers. Thus, the flour from the tuber can be used as an alternative source of calcium in food formulations and product developments.

**TABLE 2:** Mineral composition of raw, blanched, and boiled anchote flours dried at different drying temperatures

PT	DT (^o^C)	Mineral composition (mg/100g)				
		Ca	Mg	Fe	Zn	Cu
Raw	60	436.21 ± 4.56^c^	202.18 ± 0.32^b^	14.20 ± 0.55^c^	2.20 ± 0.12^c^	2.13 ± 0.13^ab^
	80	482.89 ± 5.52^a^	202.85 ± 11.76^b^	10.87 ± 0.87^d^	2.44 ± 0.27^b^	2.25 ± 0.05^a^
	100	475.70 ± 14.63^a^	222.38 ± 9.56^a^	14.13 ± 0.17^c^	2.80 ± 0.13^a^	2.10 ± 0.14^ab^
Blanched	60	414.72 ± 6.60^d^	167.39 ± 9.75^c^	14.49 ± 0.55^bc^	2.22 ± 0.10^c^	1.94 ± 0.04^b^
	80	409.58 ± 19.00^d^	160.30 ± 22.14^cd^	13.75 ± 0.28^c^	2.41 ± 0.06^b^	1.90 ± 0.34^b^
	100	462.62 ± 2.46^b^	154.93 ± 17.73^d^	14.88 ± 0.95^b^	2.69 ± 0.27^a^	2.05 ± 0.21^ab^
Boiled	60	357.07 ± 2.58^f^	158.07 ± 8.39^cd^	15.97 ± 0.15^a^	2.15 ± 0.04^c^	1.98 ± 0.16^b^
	80	359.94 ± 1.92^f^	138.87 ± 2.18^e^	13.88 ± 0.24^c^	2.19 ± 0.18^c^	1.93 ± 0.5^b^
	100	386.18 ± 3.30^e^	139.24 ± 3.66^e^	16.00 ± 0.10^a^	2.07 ± 0.05^c^	2.01 ± 0.17^ab^
LSD (0.05)		8.71	11.52	0.52	0.16	0.24
CV (%)		2.09	6.77	3.35	6.73	8.57

Note

Data are mean ± standard deviation of triplicate determinations. Means with same letters in a column are not significantly different (*p* < .05).

Abbreviations: Ca, calcium; Cu, copper; CV, coefficient of variation; DT, drying temperature; Fe, iron; LSD, least significant difference; Mg, magnesium; PT, predrying treatment; Zn, zinc.

#### 3.2.2 Magnesium (Mg)

According to the present study, the concentration of Mg in the raw, blanched, and boiled anchote flours dried at different temperatures ranged between 138.87 and 222.38 mg/100 g (Table 2). Similar to the ash content result, the effect of drying temperature was not significant (*p* > .05) on Mg content; however, blanching and boiling showed a significant negative effect (*p* < .05) on the Mg content. The value of Mg content in the present result is relatively higher than the previous report of Fekadu (2014) in which 79.73 and 73.50 mg/100 g for raw and boiled anchote, respectively. Even much lower values reported in the work of Parmar et al. (2017) and Ayalew (2016) for different anchote accessions. However, when compared with other root and tuber crops, Mg concentration of anchote flour is relatively lower than sweet potato flour (49.37–540.87 mg/100 g) (Koua et al., 2018). On the other hand, it is by far higher than potato (21 mg/100 g) and taro (7.24 mg/100 g) (Adane et al., 2013; Rahman et al., 2015). In conclusion, the raw anchote flour could be used as a good source of dietary Mg.

#### 3.2.3 Iron (Fe)

The Fe content of raw and pretreated anchote flours ranges between 10.87 and 16.00 mg/100 g (Table 2). The pretreatments (blanching and boiling) significantly (*p* < .05) enhanced Fe content, but an increase in drying temperature was not. The same trend was reported by Fekadu (2014) in which iron content of 5.49 and 6.60 g/100 g reported for raw and boiled anchote, respectively. Shebabaw (2013) also obtained a similar result in Nekemte (raw 13.38 and boiled 14.85 mg/100 g) and Dembidollo (raw 14.12 and boiled 15.63 mg/100 g) accessions. Increase in Fe content by boiling is also observed in other tuberous products like *D. bulbifera* (Re, Oduro, & Wo, 2013), taro (Adane et al., 2013; Ayele, 2009 *),* yam (Ayele, Urga, & Chandravanshi, 2015), cocoyam (Lewu et al., 2009), and Irish potato (Ikanone & Oyekan, 2014). The increase may be due to contamination from stainless steel slicers and cooking utensils (Ayele, 2009; Fekadu, 2014; Shebabaw, 2013). However, a decrease in Fe content also reported in other publications for tuber crops such as sweet potato (Ikanone & Oyekan, 2014), yam (Ayele, 2009) and taro (Ayele et al., 2015). The variation may be due to the existence of Fe in different oxidation states; Fe^3+^ is less soluble than Fe^+2^ (Ayele, 2009). The result of this study is almost similar to the report of Shebabaw (2013) for Nekemte and Dembidollo anchote accessions, whereas it was greater than Fe content reported in Fekadu (2014). Significantly higher Fe content was observed in anchote as compared with potato (0.78 mg/100 g) and taro (5.86 mg/100 g) flours (Adane et al., 2013; Rahman et al., 2015), but lower than the Fe content of yam *(Dioscorea abyssinica)* (20.3–69.7 mg/100 g) (Bekele & Bekele, 2018). Based on the result, boiled anchote flour is a better source of Fe relative to its raw form and also compared to some other tuberous crops (potato, taro, and D. bulbifera).

#### 3.2.4 Zinc (Zn)

Zinc is one of the essential micronutrients, and it is needed for the immune system of the body to function appropriately and also involved in cell division, cell growth, and wound healing (Navarre, 2009). It is among the essential nutrients to be considered in food product fortification. The average Zn content of anchote flour was affected by blanching/boiling and drying temperatures as presented in Table 2. The content ranged between 2.05 and 2.99 mg/100 g, and like other minerals, the Zn content was negatively and significantly (*p* < .05) affected by predrying treatments than drying temperature. This is comparable and in agreement with what reported in Fekadu (2014) (raw 2.23 and boiled 2.03 mg/100 g) and Ayalew (2016) (0.32–3.41 mg/100 g). In all the cases, heat treatment in water might contribute to a significant leaching loss of Zn. But results in this study are higher than Zn content of anchote (0.58 mg/100 g) as indicated in Parmar et al. (2017) and, however, relatively lower than Zn content of Nekemte (raw 6.43 and boiled 5.34 mg/100 g) and Dembidollo (raw 6.37 and boiled 5.29 mg/100 g) anchote accessions *(*Shebabaw, 2013 *)*. This might be associated with variation in agroecology zones as well as genetic differences among studied accessions. The Zn content of anchote in this study is higher compared to sweet potato and yam (Bekele & Bekele, 2018; Koua et al., 2018), but higher Zn content was reported in taro (Adane et al., 2013). Thus, it is safe to conclude that anchote would be a relatively good source of Zn, and its flour obtained from the raw tuber has preserved more Zn compared to the blanched and boiled.

#### 3.2.5 Copper (Cu)

Copper is used for the synthesis of hemoglobin, proper iron metabolism, and maintenance of blood vessels (Navarre, 2009). Table 2 shows the effect of predrying treatment and drying temperature on Cu content of anchote flours. The Cu contents of all the tuber flours were ranged in 1.65–2.30 mg/100 g with the more significant (*p* < .05) effect of predrying treatments than drying temperature. The average Cu content in the present result is higher than what reported in Ayalew (2016) (0.47–1.60 mg/100 g). Parmar et al. (2017) have also reported even further lower concentration (*0.124 mg/100 g*) in white anchote. Furthermore, the result of this study shows that Cu content of anchote is higher than potato (0.623 – 0.658 mg/100 g) and sweet potato (0.30 – 0.49 mg*/100 g*) (Baranowska, Zarzecka, Gugała, & Mystkowska, 2017; Chipungu et al., 2017). However, a relatively higher Cu content was reported in a wild tuber, *Raphionacme Splendens*, found in Sudan (3.6 mg/100 g) (Doka, El Tigani, & Yagi, 2016). Therefore, the raw anchote flour can be used as a good source of Cu compared to other commonly consumed tuber crops.

### 3.3 Thermal and physical properties

#### 3.3.1 Bulk density (BD)

The BD of raw, blanched, and boiled anchote flours dried at temperatures of 60, 80, and 100°C is presented in Table 3. The BD of the test anchote flours was ranged from 0.71 to 0.83 g/cm^3^. The result showed a significant (*p* < .05) increase in BD of the flours with pretreatments (blanching and boiling) and an increase in drying temperature. It was in agreement with the results observed in raw (0.65 g/cm^3^) and boiled (0.86 g/cm^3^) cassava flours (Kasaye, Melese, Amare, & Hailaye, 2018). There are studies showed that an increase in BD of flours associated with blanching and boiling, such as in yam (raw 0.65 and blanched 0.72 g/cm^3^) and in cocoyam (blanched 0.59 and boiled 0.63 g/cm^3^) (Akubor, 2017; Ukom et al., 2018). The BD also increased with increase in drying temperature. An increase with drying temperature could be attributed to the change in starch polymer structure to denser weight or greater reduction in flour volume as compared to its mass. Furthermore, the variation in BD could be due to differences in particle size and moisture content of the flours (Chandra, Singh, & Kumari, 2015) which might be associated with the effect of predrying treatment and drying temperature. When compared with other tuber crops, the BD of anchote flour was higher than BD of *Dioscorea dumetorum and Dioscorea bulbifera* (0.50–0.62 g/cm^3^) (Abiodun, Adegbite, & Oladipo, 2010; Kayode et al., 2017). However, it is less than the BD of yam (1.14 g/cm^3^) and cocoyam (0.94–1.01 g/cm^3^) flours (Falade & Okafor, 2015; Nina et al., 2017).

**TABLE 3:** Thermophysical properties of raw, blanched, and boiled anchote flour dried at different temperatures

PT	DT (^o^C)	Thermophysical properties			
		*BD (g/cm^3^)*	*Cp (KJ/Kg ^o^C)*	*K (W/m ^o^C)*	*α (m^2^/s)*
Raw	60	0.72 ± 0.02^e^	1.770 ± 0.007^b^	0.270 ± 0.002^ab^	2.12 × 10^–4^ ± 0.00^a^
	80	0.75 ± 0.01^d^	1.747 ± 0.003^d^	0.267 ± 0.003^bc^	2.04 × 10^–4^ ± 0.00^b^
	100	0.77 ± 0.01^c^	1.710 ± 0.003^f^	0.263 ± 0.002^d^	1.99 × 10^–4^ ± 0.00^c^
Blanched	60	0.73 ± 0.01^e^	1.767 + 0.004^b^	0.271 + 0.001^a^	2.10 × 10^–4^ ± 0.00^a^
	80	0.78 ± 0.02^c^	1.747 + 0.006^d^	0.268 + 0.002^b^	1.96 × 10^–4^ ± 0.00^cd^
	100	0.82 ± 0.01^a^	1.732 + 0.004^e^	0.266 + 0.002^bc^	1.99 × 10^–4^ ± 0.00^c^
Boiled	60	0.79 ± 0.02^bc^	1.779 ± 0.003^a^	0.272 ± 0.002^a^	1.95 × 10^–4^ ± 0.00^d^
	80	0.82 ± 0.01^a^	1.756 ± 0.016^c^	0.271 ± 0.002^a^	1.88 × 10^–4^ ± 0.00^e^
	100	0.80 ± 0.02^b^	1.780 ± 0.005^a^	0.273 ± 0.002^a^	1.92 × 10^–4^ ± 0.00^d^
LSD (0.05)		0.016	0.0057	0.0027	3.17 × 10^–6^
CV		2.08	0.33	1.01	1.60

Note

Data are mean ± standard deviation of triplicate determinations. Means with same letters in a column are not significantly different (*p* < .05).

Abbreviations: BD, bulk density; Cp, specific heat capacity; CV, coefficient of variation; DT, drying temperature; K, thermal conductivity; LSD, least significant difference; PT, predrying treatment; α, thermal diffusivity.

In the present study, the BD was relatively high (>0.7 g/cm^3^) (Falade & Okafor, 2015) in all the cases. The high BD of flour suggests that it can be used as a thickener in food products **(**Ezeocha, Oti, Chukwuma, & Aniedu, 2014). However, flours having high BD are not recommended in baby food formulations because it does not provide a nutrient‐dense meal for infants and young children (Akinsola, Idowu, Babajide, Oguntona, & Shittu, 2018; Ezeocha et al., 2014). This may also suggest alternative use of the flour as a drug binder and supporting the release of active ingredients in pharmaceutical industries (Omoniyi, Awonorin, Idowu, & Adeola, 2016). In contrast, flours with low BD would mix well during dough preparation so that they may be good functional ingredients for weaning food formulations (Akubor, 2017). This suggests that anchote flours could be suitable to be used as thickeners in food industries. The boiled anchote flour, with higher BD, is the most efficient as a thickener compared to the raw and blanched flours. The flour obtained from raw anchote dried at a lower temperature, with lower BD, may be comparatively better suited in the formulation of weaning foods.

#### 3.3.2 Specific heat capacity (Cp)

Specific heat capacity is useful information in the design of heat exchanger, choice of a heat transfer medium, and processing conditions (Ajala, Ogunsola, & Odudele, 2014). In anchote flour, the *Cp* ranged between 1.71 and 1.78 KJ/kg^o^C with boiled sample dried at 100°C having the highest value than lowest value for raw sample dried at 100°C (Table 3). Both drying temperature and predrying treatments significantly affected the *Cp* of the flours (*p* < .05). The *Cp* value of the flour decreased with an increase in drying temperature, which is in agreement with Ajala et al. (2014) for cocoyam flour. However, it was enhanced by blanching and boiling the anchote tuber before making the flour. The variation in *Cp* of the flours might be due to the moisture content difference and change in its composition by the pretreatments (Božiková, Híreš, Valach, Malínek, & Mareček, 2017). The finding showed that *Cp* of anchote flour is similar to that of cocoyam flour (1.744–1.785 kJ/kg^o^C) (Ajala et al., 2014) and flours of plantain cultivars (1.67–1.7 kJ/kg^o^C) (Barine & Victor, 2016). However, it is relatively higher than the value reported for cassava flour (1.40 –1.45 KJ/Kg^o^C) (Nwabanne, 2009). Therefore, a higher quantity of thermal energy is associated with a unit mass of the boiled anchote flour for a unit change in temperature relative to the raw, whereas the flour obtained from the raw tuber dried at 100°C with the lowest *Cp* experience low quantity of thermal energy by the same change.

#### 3.3.3 Thermal conductivity (k)

Thermal conductivity, the intrinsic ability of a material, is essential to transfer heat and to predict or control heat flux and processing time. This helps to evaluate the efficiency of a material, which in turn improves the economy of the process and enhances quality products (Ajala et al., 2014). The *K* of anchote flour, as affected by predrying treatments and drying temperature, ranged between 0.260 and 0.275 W/m^o^C (Table 3). The highest value was observed in boiled anchote flour dried at 100°C, whereas raw anchote flour dried at 100°C had the lowest. The *K* of anchote flours was significantly affected (*p* < .05) by treatment with hot water (blanching and boiling) as well as increasing drying temperature (except the boiled). The *K* increased by predrying treatments and drying at lower temperature. This decline showed an inverse relationship of *K* with drying temperature similar to the report of Ajala et al. (2014). The variation might also be due to the direct relationship of the *K* with the moisture content of the products (Nwabanne, 2009). The interaction effect of the factors showed that low *K* obtained in the raw tuber dried at higher temperature. The value of the present result is similar to the report of Ajala et al. (2014) for cocoyam tuber flour (0.266–0.271 W/m^o^C). On the other hand, it is higher than the value for cassava (0.24.W/m^o^C) (Nwabanne, 2009) and plantain (0.25 W/m^o^C) (Barine & Victor, 2016) flours. The slightly higher *K* of anchote flour compared to cassava flour suggests that heat moves relatively faster through it. The finding revealed that thermal flow was fastest in the boiled and slowest in the raw anchote flour. This implies that the value‐added anchote flour produced after predrying treatment has improved cooking performance.

#### 3.3.4 Thermal diffusivity

Thermal diffusivity is a measure of how fast the heat diffuses or the temperature spreads through a material. The speed of heat diffusion through a material is relevant information in processing time prediction (Ajala et al., 2014). The thermal diffusivity of the samples in the present study increased with a decrease in moisture content that is in agreement with the report of Nwabanne (2009). The thermal diffusivity value of anchote flours ranged between 1.86 × 10^–4^ and 2.17 × 10^–4^ m^2^/s with raw anchote dried at 60°C having the highest value and boiled anchote dried at 80°C had the lowest. The effect of hot water treatment and drying temperature on the thermal diffusivity of anchote flours was statistically significant (*p* < .05). The predrying treatments and high drying temperature decreased the thermal diffusivity. Even though it was expected to increase in the value, a decrease in *Cp*, and a significant change in BD and proportional change in *K* results in a decrease in the values. The data for anchote are comparable with thermal diffusivity of fermented cassava flour reported by Nwabanne (2009). However, it is slightly smaller than the thermal diffusivity value of cocoyam flour (2.84 × 10^–4^–4.22 × 10^–4^ m^2^/s), as reported by Ajala et al. (2014). The difference could be due to the difference in density of the flours. But the value in this study is higher than the values reported for potato (10 × 10^–8^ to 16 × 10^–8^ m^2^/s) and sweet potato tubers (10 × 10^–8^ to 12 × 10^–8^ m^2^/s), respectively (Oluwo, Khan, & Salami, 2013). That means the heat easily diffuses through anchote flour compared to potato and sweet potato. This can be an indication that its cooking property has been improved when dried and converted into flour compared to its raw tuber.

## 4 CONCLUSIONS

Anchote tuber is a nutritionally valuable but underutilized tuber crop in Ethiopia. However, the extensive utilization of the crop highly limited due to the lack of sufficient scientific pieces of evidence associated with the use of anchote tuber in different forms. This study showed that the potential use of anchote flour with minimum alteration of the composition of the flour in terms of proximate composition, minerals content, and thermophysical properties. Most of the proximate contents were high in the raw anchote flour dried at higher temperature. However, protein was more preserved in the raw flour dried at lower temperatures.

Moreover, except Fe, more minerals were retained in raw anchote flour dried at 80 or 100°C. The same is true for higher thermal diffusivity value as compared to predrying processed tubers. The contribution of both predrying treatments was less in terms of getting better quality anchote flour. Therefore, the flour obtained from the raw tuber dried at relatively higher temperatures (up to 100°C) could be recommended for better nutritional value and thermal property. This will help to use anchote tuber in different forms than the sole use of the tuber after a long time of cooking. As a rich mineral source, the flour can be used as an ingredient in different food formulations to compliment mineral deficit foods. In addition to this, the flour under good packaging and storage conditions can be stored for a longer time as compared to the highly perishable tuber. It also avoids underground storage in the field and offer opportunity to producers to use the field for other crops.

## CONFLICT OF INTEREST

The authors declare no conflict of interest.

## ETHICAL APPROVAL

This study does not involve any human or animal testing.
